# PD39 First Approach For Assessing Statistical Significance In Industry Funded Matching-Adjusted Indirect Comparison Studies: A Scoping Review

**DOI:** 10.1017/S0266462324003027

**Published:** 2025-01-07

**Authors:** Cecilia Farinasso, Aline da Rocha, Flávia Medeiros, Lays Marra, Patricia Parreira, Layssa Oliveira, Vinicius Ferreira, Rosa Lucchetta, Haliton Alves De Oliveira Junior

## Abstract

**Introduction:**

When indirectly compared trials are too heterogeneous to provide a reliable estimate, matching-adjusted indirect comparison (MAIC) studies can be employed. This technique is commonly used for oncology treatments. MAIC is an indirect comparison that adjusts effect-modifying variables through propensity score methods. The objective of this study was to map the characteristics of MAIC studies in oncology.

**Methods:**

We performed a scoping review of the characteristics of MAIC studies that applied MAIC to compare active treatments in oncology. The literature search was last updated in August 2023 in PubMed, Embase, and the Cochrane Library. We extracted sources of funding, outcomes reported, and whether the results were significantly in favor of the trial for which individual patient data (IPD) were available or for the aggregate data. We then calculated the relative risk (RR) and confidence interval (CI) of an outcome favoring the IPD trial technology that was also funded by industry.

**Results:**

A total of 90 studies were included in the review. The pharmaceutical industry was the most frequent funder (n=78; 87%); the source of the IPD data was not reported in 68 studies (76%). In total, 391 efficacy outcome estimates were reported in base case analyses. The risk of favoring IPD while being funded by industry was 93 percent, while the risk of favoring IPD while having other sources of funding was 61 percent (RR 1.520, 95% CI: 1.146, 2.016; p=0.004). Specifically, the RR was 1.246 (95% CI: 0.891, 1.743) for overall survival and 1.426 (95% CI: 0.959, 2.120) for progression-free survival.

**Conclusions:**

MAIC results are influenced by the choice and number of effect-modifying variables used for matching the population. National Institute for Health and Care Excellence guidelines consider it necessary to provide evidence that the matched estimate will be less biased than the unmatched one. We have concluded that industry funded MAIC studies may be more likely to report results favoring IPD than studies with another funding source.

